# Supplementary material for: Genetic testing offer for inherited neuromuscular diseases within the EURO-NMD reference network: A European survey study
Source: PLoS One. 2020 Sep 18;15(9):e0239329. doi: 10.1371/journal.pone.0239329 (PMC7500674; doi:10.1371/journal.pone.0239329)
Supplement: S1 Table — (PDF) [file pone.0239329.s001.pdf]

# Section A: HCP Information

**A1. Are you a member of EURO-NMD? If so please select your HCP from the dropdown menu.**

- HCP01 - JWMDRC, Newcastle University ☐
- HCP02 - Cliniques Universitaires de Bruxelles - Hopital Erasme ☐
- HCP03 - University Hospital of Saint-Etienne ☐
- HCP04 - UZ Gent ☐
- HCP05 - Antwerp University Hospital (UZA) ☐
- HCP06 - University Hospitals Saint-Luc ☐
- HCP07 - Expert Centre for Hereditary, Neurologic and Metabolic Disorders ☐
- HCP08 - Motol University Hospital ☐
- HCP09 - University Hospital Brno, Neuromuscular Centre ☐
- HCP10 - Tampere University Hospital ☐
- HCP11 - Assistance Publique - Hopitaux de Paris (APHP) Consortium (NeMusChALS) ☐
- HCP12 - Hôpital Bicêtre, Hôpitaux universitaires Paris-Sud, Assistance Publique – Hôpitaux de Paris ☐
- HCP13 - Centre Hospitalier (Univeritaire de Nice (CHUN)) ☐
- HCP14 - Assistance Publique -Hopitaux de Marseille ☐
- HCP15 - CHU Limoges ☐
- HCP16 - APHP Raymond Poincaré Hospital, University Hospitals Paris-Ouest ☐
- HCP17 - Nantes University Hospital ☐
- HCP18 - Charité-Universitätsmedizin Berlin ☐
- HCP19 - University Hospital of Bonn ☐
- HCP20 - Neuromuscular Center of the University Medical Center Gottingen ☐
- HCP21 - Universitätsklinikum Ulm (UKU) in cooperation with the Universitätsund Rehabilit ☐
- HCP22 - University Hospitals Leuven ☐
- HCP23 - Friedrich-Baur Institute ☐
- HCP24 - Childrens Clinic Essen University Hospital ☐
- HCP25 - Dr V Hauner Children's Hospital, Ludwig-Maximillians-University ☐
- HCP26 - University of Pecs ☐

▼

HCP27 - Semmelweis University ☐

HCP28 - AOU Policlinico "G.Martino" Messina ☐

HCP29 - Ospedale Pediatrico Bambino Gesù IRCCS ☐

HCP30 - Istituto Nazionale Neurologico Carlo Besta ☐

HCP31 - Azienda Ospedaliera Padova ☐

HCP32 - Fondazione Policlinico Universitario A.Gemelli ☐

HCP33 - Azienda Ospedaliera Universitaria Senese ☐

HCP34 - Azienda Ospedaliera-Universitaria Ferrara ☐

HCP35 - Azienda Ospedaliero-Universitaria Pisana ☐

HCP36 - AOU - Second University of Naples (SUN) ☐

HCP37 - AOU - ASST "Spedali Civili" ☐

HCP38 - Fondazione IRCCS Ca' Granda Ospedale Maggiore Policlinico Milano ☐

HCP39 - Paediatric Neurology and Neuromuscular Disorders Unit -Gaslini Institute ☐

HCP40 - Azienda Ospedaliera Universitaria Citta della Salute e della Scienza di Torino ☐

HCP41 - Nemo Clinical Center (Neuromuscular Omnicomprehensive) ☐

HCP42 - Istituto Auxologico Italiano Istituto di Ricovero e Cura a Carattere Scientifico ☐

HCP43 - Academic Medical Centre ☐

HCP44 - Erasmus MC University Medical Center ☐

HCP45 - Radboud University Medical Center ☐

HCP46 - Maastricht UMC+ ☐

HCP47 - Leiden University Medical Center ☐

HCP48 - Medical Center - University of Freiburg ☐

HCP49 - University Medical Centre Utrecht, Section Neuromuscular Diseases ☐

HCP50 - Department of Neurology, Medical University Teaching Hospital (SPCSK) ☐

HCP51 - University Medical Center Ljubljana ☐

HCP52 - Hospital Sant Joan de Déu ☐

HCP53 - Complejo Hospitalario Regional Virgen del Rocío ☐

HCP54 - Hosipital de la Santa Creu i Sant Pau ☐

HCP55 - Hospital Universitari Vall D'Hebron ☐

▼

HCP56 - Hospital UiP La Fe ☐

HCP57 - Sahlgrenska University Hospital ☐

HCP58 - Karolinska Universitetssjukhuset ☐

HCP59 - University College London Hospitals ☐

HCP60 - Great Ormond Street Hospital, Foundation Trust ☐

HCP61 - Oxford Neuromuscular Centre ☐

**A2. If NO, are you a member of any other ERNs? If so please select your Network(s) from the list.**

ERN BOND (European Reference Network on bone disorders) ☐

ERN CRANIO (European Reference Network on craniofacial anomalies and ear, nose and throat (ENT) disorders) ☐

Endo-ERN (European Reference Network on endocrine conditions) ☐

ERN EpiCARE (European Reference Network on epilepsies) ☐

ERKNet (European Reference Network on kidney diseases) ☐

ERN-RND (European Reference Network on neurological diseases) ☐

ERNICA (European Reference Network on inherited and congenital anomalies) ☐

ERN LUNG (European Reference Network on respiratory diseases) ☐

ERN Skin (European Reference Network on skin disorders) ☐

ERN EURACAN (European Reference Network on adult cancers (solid tumours)) ☐

ERN EuroBloodNet (European Reference Network on haematological diseases) ☐

ERN eUROGEN (European Reference Network on urogenital diseases and conditions) ☐

ERN EYE (European Reference Network on eye diseases) ☐

ERN GENTURIS (European Reference Network on genetic tumour risk syndromes) ☐

ERN GUARD-HEART (European Reference Network on diseases of the heart) ☐

ERN ITHACA (European Reference Network on congenital malformations and rare intellectual disability) ☐

MetabERN (European Reference Network on hereditary metabolic disorders) ☐

ERN PaedCan (European Reference Network on paediatric cancer (haemato-oncology)) ☐

ERN RARE-LIVER (European Reference Network on hepatological diseases) ☐

ERN ReCONNET (European Reference Network on connective tissue and musculoskeletal diseases) ☐

ERN RITA (European Reference Network on immunodeficiency, autoinflammatory and autoimmune diseases) ☐

ERN TRANSPLANT-CHILD (European Reference Network on Transplantation in Children) ☐

VASCERN (European Reference Network on Rare Multisystemic Vascular Diseases) ☐

# Section B: Main Survey

**B1.** **1. How many genetic tests (of all kinds) for Neuromuscular Diseases (NMDs) do you perform in your centre per year?**

- More than 5,000 ☐
- 1000 - 5000 ☐
- 500 - 1000 ☐
- 100 - 500 ☐
- Less than 100 ☐

**B2.** **2. Is your laboratory registered on the Orphanet database?**

- Yes ☐
- No ☐

**B3.** **2a. If you answered YES to Q2, please provide your Centre's EUGT number.**

**B4.** **2b. If you answered NO to Q2, please list the NMDs for which your Centre provides genetic tests**

**B5.** **2c. If you do not have or cannot provide the EUGT number please select from the list below those tests which are performed at your Centre.**

- Panels of selected genes ☐
- Exome sequencing - panels are selected bioinformatically ☐
- Exome sequencing - target genes are defined by HPO terms ☐
- We systematically use trio design (affected person, mother, father) for most of our NGS testing ☐
- Whole genome sequencing ☐
- RNAseq ☐
- Other ☐

Other

**B6.** **3. In your Centre, which are the most commonly used technologies for NMD diagnosis?**

- Sanger sequencing ☐
- MLPA ☐
- Southern-blotting ☐
- NGS ☐

Other ☐

Other

[ ]

**B7.** **4. What share does NGS represent in comparison to targeted gentic testing (Sanger sequencing/other targeted genetic tests) for NMDs in your Centre?**

- More than 50% ☐
- 10%-50% ☐
- 1%-10% ☐
- Less than 1% ☐

**B8.** **5. If your Centre uses an NGS approach in the diagnosis of NMDs, please specify**

- You use NGS for all patients that fulfil diagnostic criteria for the NMD which would profit from NGS testing ☐
- You use NGS for selected patients (i.e. evidence of positive family history, if the targeted genetic testing was negative ...) ☐
- You use NGS, but only in the research setting ☐
- You do not use NGS in your Centre ☐
- Other (please specify) ☐

[ ]

**B9.** **6. What is the coverage of genetic tests that are currently available for rare NMDs in your country?**

- Genetic tests for most NMDs are provided by national genetic services ☐
- National genetic services which provide genetic testing for some disorders, abroad genetic testing is organised for disorders not covered by our National Health system ☐
- Access to genetic testing for NMDs in my country is limited due to lack of genetic testing for the full spectrum of NMDs and is most pertinent for the following NMDs.... (please specify opposite) ☐

**B10.** **7. What is your Centre's approach to likely pathogenic Variants of Uncertain Significance (VOUS)?**

- [ ] We close the case with a report, in terms of routine diagnostic evaluation
- [ ] We re-evaluate all cases in defined time intervals (i.e. annually)
- [ ] We systematically use matchmaking options
- [ ] Other (please specify opposite)

**B11.** **8. Does your Centre perform routine Sanger sequencing validation of NGS identified variants?**

- [ ] Yes
- [ ] No

**B12.** **9. What is your Centre's approach for reporting incidental findings?**

- [ ] We use panel testing, therefore the level of incidental findings is very low
- [ ] We use exome sequencing and we are obliged to report predispositions for serious, treatable disorders to all the patients
- [ ] We use exome sequencing and offer all patients the option to learn predispositions for serious, treatable disorders
- [ ] We use exome sequencing and we do not offer an option to learn predispositions for serious, treatable disorders
- [ ] Other (please specify)

**B13.** **10. What is your experience with NGS?**

- [ ] NGS has improved the diagnostic yield in our Centre
- [ ] NGS has improved access to genetic testing
- [ ] So far, no impact of NGS has been noticed
- [ ] NGS is still in a translational phase - it is too early to assess the impact
- [ ] Other

Other

**B14.** **11. What is the clinical pathway to refer a patient for NGS testing in your Centre?**

- [ ] Neurologist refers a patient's sample directly to the NGS laboratory
- [ ] Neurologist refers a patient's sample to a clinical/medical geneticist who decides on NGS testing
- [ ] Neurologist refers patients for pre-test genetic counselling
- [ ] There is a multidisciplinary team, which decides on NGS testing
- [ ] Other

Other

**B15.** **12. How does your Centre prioritise NGS in diagnostic algorithms?**

- [ ] NGS is performed as a first tier genetic diagnostic tool if there is a diagnostic hypothesis of NMD with genetic etiology (not associated with a single gene or specific mutational mechanism, i.e. deletion in SMA or expansion in Kennedy disease)
- [ ] NGS is performed only after traditional diagnostic workup (incl. muscle biopsy, imaging)
- [ ] Other (please specify)

**B16.** **13. What are the barriers for NGS implementation in your country?**

- [ ] Limited evidence of benefit/value

Lack of awareness/acceptance among neurologists ☐

Lack of experience/equipment for NGS provision in the country ☐

Difficulties in implementing cross-border genetic testing ☐

Lack of guidelines/clinical pathways ☐

Lack for reimbursement ☐

Other ☐

Other

**B17. 14. What activities could contribute to more efficient implementation of NGS in the future?**

Education for neurologists & associated health professionals ☐

Standardisation of NGS procedures ☐

International databases of pathologic gene variants for NMDs ☐

International form to evaluate cases with negative NGS results ☐

Implementation of new NGS applications including Whole Genome Sequencing, RNAseq ☐

Other ☐

Other

**B18. 15. Does your centre perform in-house bioinformatics analysis?**

Yes ☐

No ☐

**B19. 16. Does your Centre participate in External Quality Assessment schemes for NMDs?**

Yes ☐

No ☐

**B20.** **17. Does your Centre participate in the External Quality Assessment scheme for NGS?**

Yes ☐

No ☐

**Thank you for your participation, your contribution is greatly appreciated.**
